# Impact of Uniaxial Stretching on Both Gliding and Traction Areas of Tendon Explants in a Novel Bioreactor

**DOI:** 10.3390/ijms21082925

**Published:** 2020-04-22

**Authors:** Mersedeh Tohidnezhad, Johanna Zander, Alexander Slowik, Yusuke Kubo, Gözde Dursun, Wolfgang Willenberg, Adib Zendedel, Nisreen Kweider, Marcus Stoffel, Thomas Pufe

**Affiliations:** 1 Anatomy and Cell Biology, Uniklinik RWTH Aachen University, Wendlingweg 2, 52074 Aachen, Germany; johanna.bartsch@rwth-aachen.de (J.Z.); ykubo@ukaachen.de (Y.K.); nkweider@ukaachen.de (N.K.); tpufe@ukaachen.de (T.P.); 2 Institute of Neuroanatomy, Uniklinik RWTH Aachen University, Wendlingweg 2, 52074 Aachen, Germany; aslowik@ukaachen.de (A.S.); azendedel@ukaachen.de (A.Z.); 3 Institute of General Mechanics, RWTH Aachen University, Templergraben 64, 52056 Aachen, Germany; dursun@iam.rwth-aachen.de (G.D.); Wolfgang.Willenberg@rwth-aachen.de (W.W.); stoffel@iam.rwth-aachen.de (M.S.)

**Keywords:** gliding tendon, traction tendon, bioreactor, cyclic stretching, rupture

## Abstract

The effects of mechanical stress on cells and their extracellular matrix, especially in gliding sections of tendon, are still poorly understood. This study sought to compare the effects of uniaxial stretching on both gliding and traction areas in the same tendon. Flexor digitorum longus muscle tendons explanted from rats were subjected to stretching in a bioreactor for 6, 24, or 48 h, respectively, at 1 Hz and an amplitude of 2.5%. After stimulation, marker expression was quantified by histological and immunohistochemical staining in both gliding and traction areas. We observed a heightened intensity of scleraxis after 6 and 24 h of stimulation in both tendon types, though it had declined again 48 h after stimulation. We observed induced matrix metalloproteinase-1 and -13 protein expression in both tendon types. The bioreactor produced an increase in the mechanical structural strength of the tendon during the first half of the loading time and a decrease during the latter half. Uniaxial stretching of flexor tendon in our set-up can serve as an overloading model. A combination of mechanical and histological data allows us to improve the conditions for cultivating tendon tissues.

## 1. Introduction

### 1.1. Tendon and Clinical Problem

Tendons consist of hypovascularized and hypocellular tissue with low regeneration potential. Tendon disorders due to overuse or age-related degeneration are a common clinical problem in orthopedic medicine [1,2]. Tendon rupture is generally categorized either as spontaneous, traumatic, or open-incision. Spontaneous rupture results from overstretching and is accompanied by pathological changes in the tissue [3,4]. Treating a ruptured tendon is a lengthy process. The formation of new fibrovascular tissue with reduced stability (and consequently, reduced mechanical performance) results in a high risk of re-rupture of the tendon. Most tendon ruptures are localized in either the intrasynovial area of flexor tendons, where the blood supply to the fibrocartilaginous area is diminished within an enthesis, or where the tendon changes its tension direction (called a gliding tendon) [5,6].

**Figure 1:**
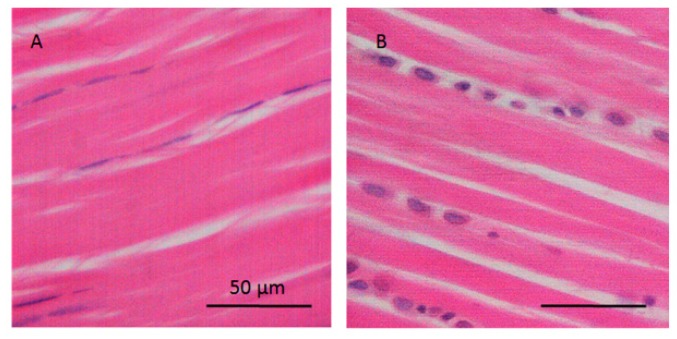
Histological images of the traction and gliding area of tendon. (**A**) Traction region with elongated tenocytes parallel to the collagen fibers and (**B**) gliding area with chondrocyte-like cells at regions of pressure in hypomochlion (tendon of flexor digitorum longus; rat).

Gliding areas of tendon differ from traction sections in that the direction of muscle contraction is not the same as the direction in which the forces are transmitted from tendon to bone. The direction of force pivots around a bony or fibrous hypomochlion. The gliding area of the flexor digitorum longus is macroscopically visible with its own tendon sheath [7]. Gliding tendons are subject to forces of shear and pressure in addition to those caused by tension. Histological analysis of the gliding area of tendon tissue indicates large, chondrocyte-like cells inside the fibrocartilage and high levels of acidic glycosaminoglycans in the extracellular matrix (ECM) [8] (see Figure 1). Gliding tendon exhibits less vascularization and reduced tensile strength, but greater compressive strength, than traction regions of the tendon. The different mechanical characteristics of tendons depend on their type, structure, thickness, and stiffness. In fibrocartilaginous tissue, such as the gliding region of tendon, strain transfer from the muscle to the cells and ECM of the inhomogeneous fibrocartilaginous tissue is the primary cause of underlying fibrous structure being heterogenic [9]. This structural property may play an important role in how a tendon responds to fatigue loading and in the healing process. A further purpose of this study was to determine how cyclic uniaxial loading of tendon affects protein expression in the tenocytes of both tendon types. 

The chronic overloading of tendon induces matrix degradation because of increasing MMPs and collagen degradation markers [10]. Mechanical overload upregulates vascular endothelial growth factor (VEGF) expression in tenocytes. Studies have found high VEGF expression in degenerative tendons [8,11]. VEGF expression is accompanied by the expression of matrix metalloproteinases (MMPs), and it inhibits the expression of tissue inhibitors of metalloproteinase (TIMP) in various cell types and thus the reduction of tissue resistance [11,12,13]. 

In complex injuries, tendon grafts may be indicated to restore gliding function. Currently, traction tendons, being readily available and relatively easy to harvest, are the most common source of tendon autografts [14,15,16]. However, these donor tendons do not match the functional or anatomical characteristics of the fibrocartilaginous flexor tendons that they are intended to replace [6]. Many studies have investigated the pathomechanism underlying tendon injuries. However, the pathophysiology of tendon injuries is nevertheless still not clearly understood [17,18]. Treatment strategies for tendons, including gliding areas, are limited by the scant evidence surrounding their inhomogeneous tissue structure and cellular response to mechanotransducive processes. 

### 1.2. The Impact of Mechanical Loading

The vertebral musculoskeletal system relies on mechanical loading. Tendons are specialized connective tissues that connect and transmit forces from muscle to bone. Thus, they enable joint movements. They can store elastic energy and withstand the high tensile forces upon which locomotion is entirely dependent. 

Normally, mature tendons are characterized by low cellular density. Tenocytes can synthesize all components of the tendon matrix, with peak activity occurring during growth, and gradual decrease during aging [19]. The extracellular matrix (ECM) of tendon tissue is composed of collagen fibers (types I and III, with low levels of types V, VI, XII, XIV, and XV) and elastic fibers. In general, elastic fibers ensure tissue flexibility and extensibility. They permit long-range deformability as well as energy storage and elastic recovery within a muscle–tendon complex. Besides the fibers, tendon ECM has many other components, including ground substance containing sulfated glycosaminoglycans (sGAGs), proteoglycans (decorin, biglycan, lumican, and fibromodulin), and structural glycoproteins, as well as a wide variety of other small molecules [20,21]. Tenocytes interact directly with the ECM and collagen fibrils. 

Tendon tissue structure allows for its typical biomechanical behavior and stress–strain curve. Elastic fibers ensure tissue flexibility, extensibility, and elastically recovery within a muscle–tendon complex. The inherent efficiency of various extremity tendons at storing elastic energy (based on their material features and characteristics) is found just as well in different-sized animals. A low tendon strain, i.e., of less than 1%–2%, is distinctive of the elastic toe region, where the crimped tendon fibers are stretched out due to mechanical loading on the tendon. A higher strain of up to 4% represents the linear region of tendon with viscoelastic recovery. In this region, the collagen fibrils are oriented in the same direction as the mechanical tensile load. Strain above 4% results in micro-tear failure of collagen fibers and sets up a micro-fatigue region. As strain increases further, up to 8%–10%, macroscopic failure and tendon injuries become detectable [2]. The mechanical characteristics of the tendon are relatively uniform across a range of vertebrate animals, such as rabbit or rat [22,23], and therefore animal models are often used in the field of tendon pathology and repair. The choice of animal depends on the modalities of the mechanical loading of the tendon [24]. Presently, based on the knowledge of tendon fatigue leading to tendinopathy, and potentially to tendon rupture, rat and mice tendons were used in the development of an in vivo fatigue model [25]. 

The mechanical loading of ECM, like tensile strain and the number and frequency of cycles, seems to be essential for maintaining tendon and tenocyte-phenotypic stability [26]. In vitro studies have shown that cyclic loading upregulates tenocyte gene expression in primary cells, tendon progenitor or stem cells, or cell-seeded bioartificial tendons as compared to static cultivation [27,28,29]. Strain stress between 4% and 10% (depending on the number of cycles and loading frequency) is shown to elicit the differentiation of tendon-derived stem cells or to upregulate the tenocyte anabolic factors tenomodulin and scleraxis [30,31,32]. Scleraxis (Scx), a member of the Twist-family of basic helix-loop-helix (HLH) transcription factors, is required for tenogenesis. This factor is shown to be critical for the development of functional tendons because it regulates tenocyte specific factors such as tenomodulin (Tnmd) and proteoglycans [33,34,35]. Scx directly transactivates Tnmd via preferential binding to specific DNA sequences via E-boxes such as SCX/E12 or SCX/E47 heterodimers [36]. The translocation of this factor into the nucleus provides evidence of its binding and activation. Scx has also been shown to regulate the expression of bone morphogenetic protein-4 in tendon cells at the fibrocartilaginous enthesis in tendon [37]. 

Most previous in vitro studies have analyzed tenocytes or fibroblasts isolated from traction tendons or ligaments [38]. The presence of fibrocartilaginous tissue in tendon has been more thoroughly explored in tendon–bone junctions. We have only scant data about the pathomechanism of gliding tendons [15,39,40]. Additionally, the effects of mechanical stress on cells and on the ECM (especially in gliding regions of tendon), as well as the regulation of the various intracellular processes, have so far not been sufficiently investigated [41,42]. The present study aims to compare the mechanical–biological interaction between tenocytes in traction and in gliding tendon tissue under cyclic uniaxial loading.

We hypothesized that the uniaxial mechanical loading has a different effect on protein expression in tenocytes in traction regions and chondrocyte-like cells in gliding regions. The signs of fatigue are seen in the composition of ECM and an increase of MMPs protein expression.

## 2. Results

Our first step was to examine the cell viability of tendon explants for 48 h in both the traction and the gliding sections during the experiments. As a negative control, we used tendon treated with H_2_O_2_ (see Supplementary Figure S1). There was no reduction of cell viability detected in either region. The gliding-area cells show a tendency toward greater activity than the traction-area cells at both 0 h and 48 h. Cell activity after treatment with H_2_O_2_ was significantly reduced in both areas. 

Next was to examine how our applied strain/stretch in the in vitro bioreactor would affect cell apoptosis. To establish a sufficient treatment protocol, we performed caspase-3 stainings of both traction and gliding regions of tendons, as well as of placental tissue as a positive control for apoptotic cells (Supplementary Figure S2). While many cells were in an apoptotic state in the placental tissue, we only occasionally observed caspase-3-positive cells in both tendon types after 48 h of uniaxial stretching. 

### 2.1. Effect of Uniaxial Treatment on sGAG Levels in Traction and Gliding Areas of Tendons

**Figure 2:**
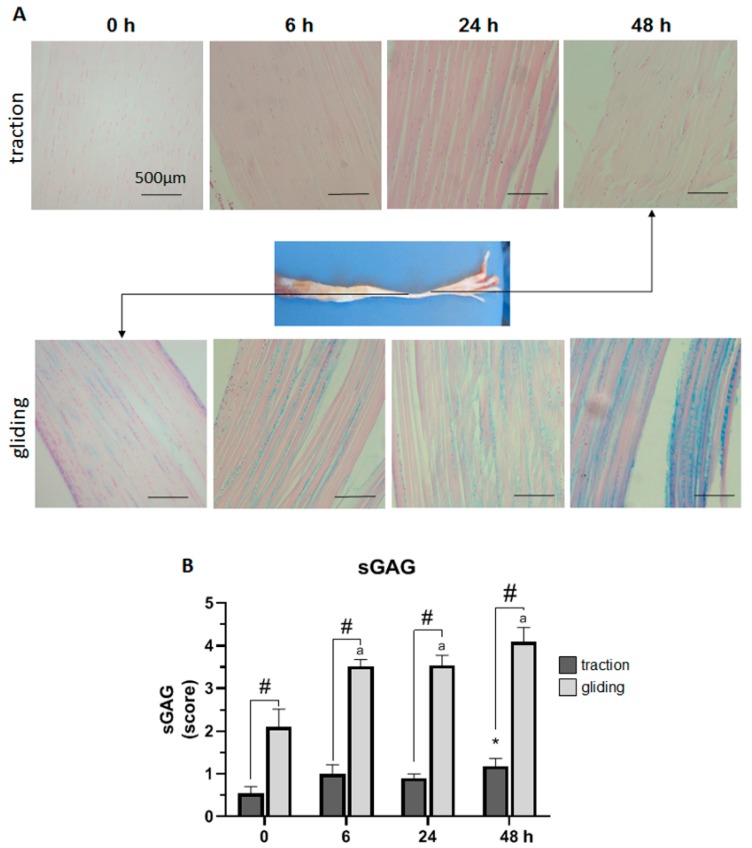
Impact of uniaxial force on sulfated glycosaminoglycan (sGAG) expression in traction and gliding areas of tendon. The images (**A**) show representative sections of the traction (upper panel) and gliding (lower panel) tendons with Alcain blue staining after uniaxial treatment. The overview shows where these tendon types are located on the flexor digitorum longus muscle. Scoring of the staining revealed a steady increase in sGAG expression in both tendon tissue types. Note that sGAG expression in the traction region showed a significant increase after 48 h (**B**); but in the gliding region, a faster response was observable after 6 h, which was then followed by a less marked increase to the highest value after 48 h. In general, sGAG expression was significantly higher in the gliding than in the traction tendon. The *p*-values are given as *p* ≤ 0.05, *p* ≤ 0.01, and *p* ≤ 0.001. Asterisks indicating statistical differences in comparison to the 0 h control time point within the traction group, letters indicate statistical difference in comparison to the control time point within the gliding group, and hashtags indicate traction vs. gliding within the different time points.

Next, we investigated the effect of the uniaxial tensile force during controlled displacement and stretching by scoring the expression of sGAG in Alcian blue-stained sections of both traction and gliding tendons (see Figure 2). To rate sGAG expression, the resulting blue staining was scored from 0 (no blue staining) to 5 (intense blue staining) and graphed as shown in Figure 2B. The uniaxial stretching of the traction region of tendon slightly increased the expression of sGAG to a significant level after 48 h of stretching. By contrast, more intense Alcian blue staining in the gliding tendon was found before the experiment, indicating a higher base expression of sGAG there. Uniaxial stretching significantly increased the expression of sGAG during the first 6 h of stretching and further after 48 h. Note that after 48 h of stretching, the expression of sGAG in both tendon types had doubled, and was in general significantly higher, in the gliding tendon.

### 2.2. Effect of Uniaxial Treatment on Scleraxis and Type-1 Collagen Protein Expression in Traction and Gliding Areas of Tendons

For our next set of experiments, we used immunofluorescence staining to examine how the uniaxial stretching force we applied affected the expression of the factors typically expressed in tendon tissue, such as Scx and Col1, and their translocation into the nucleus. 

**Figure 3:**
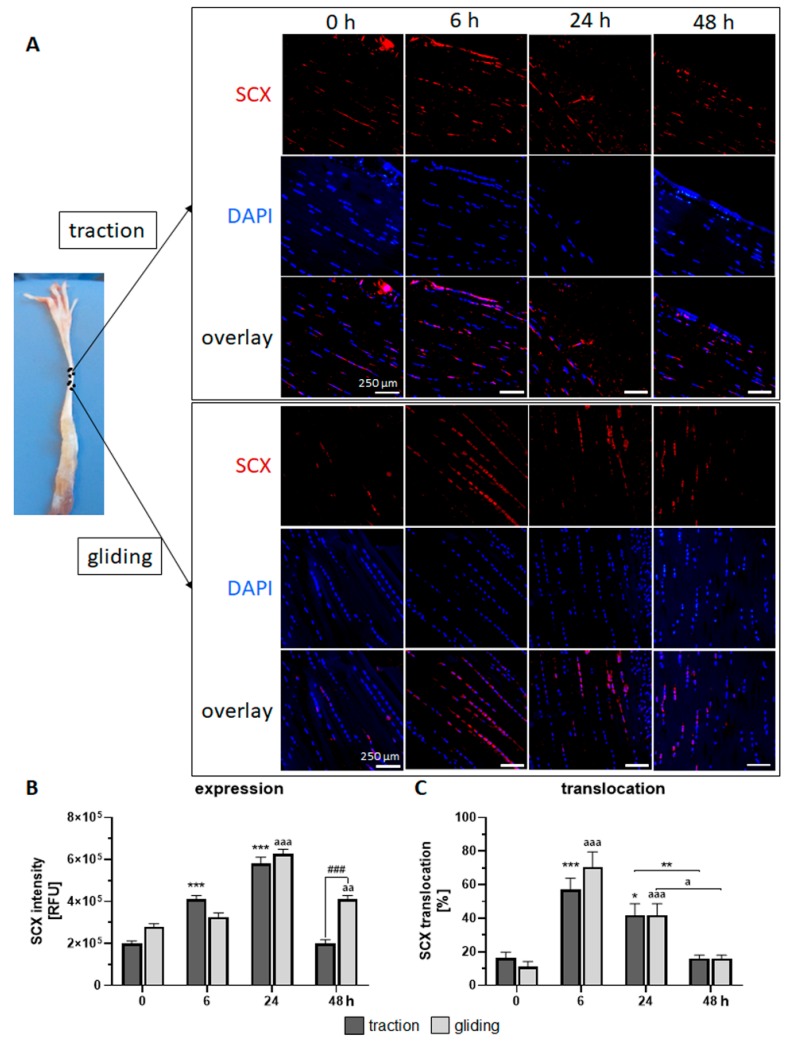
Effects of uniaxial treatment on scleraxis (Scx) expression and translocation in the traction and gliding areas of tendon. (**A**) Representative immunofluorescence-stained sections of Scx (red), DAPI (blue, nuclear staining), and a representative overlay where these tendons are located (left panel). (**B**) The application of uniaxial force increased Scx protein expression during the first 24 h in the traction region, which then returned to the basal level. In the gliding tendon, Scx expression was delayed but also peaked at 24 h before returning to basal levels. Note that after 48 h, Scx expression was higher in the gliding tendon. (**C**) Scx translocation into the nucleus occurred during the first 6 h, followed by a steady return to basal levels. The *p*-values are given as *p* ≤ 0.05, *p* ≤ 0.01, and *p* ≤ 0.001. Asterisks indicating statistical differences in comparison to the 0 h control time point within the traction group, letters indicate statistical difference in comparison to the control time point within the gliding group, and hashtags indicate traction vs. gliding within the different time points.

In the traction region (see Figure 3A upper panel, B), the expression of the Scx protein peaked after 24 h of stimulation but decreased to control levels after 48 h. Scx expression was paralleled by a high translocation rate of Scx into the nucleus during the first 6 h of uniaxial treatment, followed by a normalization to control levels (see Figure 3C). Scx expression in the gliding tendon also peaked after 24 h (but showed a delayed increase) and returned to control levels after 48 h of uniaxial treatment (see Figure 3A lower panel, B). Note that after 48 h, Scx expression was lower in the traction region. Both tendon types exhibited similar Scx translocation into the nucleus (Figure 3C). 

### 2.3. Effect on Uniaxial Treatment on the Expression of Matrix Metalloproteinases 

**Figure 4:**
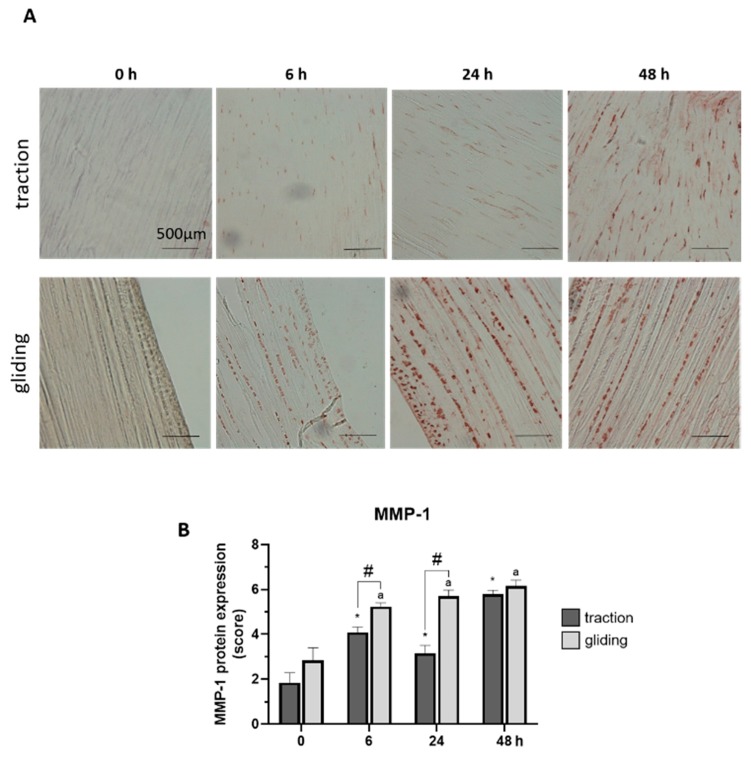
Effects of uniaxial treatment on matrix metalloproteinase-1 (MMP-1) expression in traction and gliding areas of tendons. (**A**) Representative images of immunohistochemically stained sections against MMP-1 in traction (upper panel) and gliding (lower panel) regions after 48 h of uniaxial force application. (**B**) Scoring of MMP-1 in the traction region revealed a significant increase after 6 h with a further elevation of MMP-1 expression out to 48 h. MMP-1 expression was significantly elevated after 6 h in gliding area and then slightly increased to a maximum at 48 h of treatment. Note that while peak expression was similar, expression at the 6 and 24 h time points differed in both areas. The *p*-values are given as *p* ≤ 0.05, *p* ≤ 0.01, and *p* ≤ 0.001. Asterisks indicating statistical differences in comparison to the 0 h control time point within the traction group, letters indicate statistical difference in comparison to the control time point within the gliding group, and hashtags indicate traction vs. gliding within the different time points.

**Figure 5:**
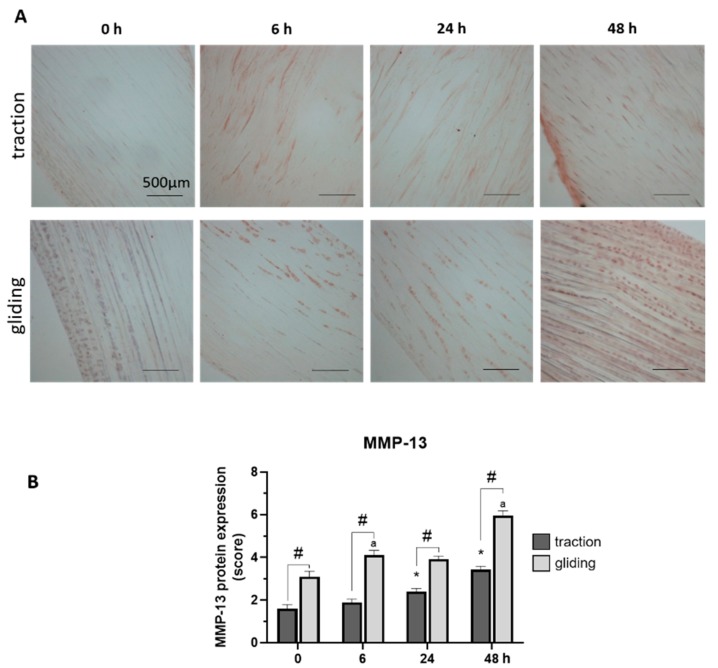
Effects of uniaxial treatment on matrix metalloproteinase-13 (MMP-13) expression in traction and gliding tendons. (**A**) Representative images of immunohistochemically stained sections against MMP-13 in traction (upper panel) and gliding (lower panel) region after 48 h of uniaxial force application. (**B**) Scoring of MMP-13 in the traction region revealed a slight increase in the expression of MMP-13; its highest expression came about at 48 h of treatment. A similar increase was observed in the gliding tendon. Note that MMP-13 exhibited a slower protein expression response to the application of uniaxial force than MMP-1 did. Further, MMP-13 expression was higher in the gliding tendon during the whole experiment. Asterisks indicating statistical differences in comparison to the 0 h control time point within the traction group, letters indicate statistical difference in comparison to the control time point within the gliding group, and hashtags indicate traction vs. gliding within the different time points.

Our last set of experiments examined the influence of applied uniaxial force on the protein expression of MMP-1 and -13 in both tendon types using immunohistochemically stained sections. Expression was evaluated by scoring of the stained areas in the tissue. The applied force increased MMP-1 in the traction-region tissue, where it peaked at 48 h of treatment (see Figure 4A,B), but in the gliding tendon tissue, MMP-1 protein expression was significantly elevated after the first 6 h time-interval but increased only slightly to its peak level at 48 h (see Figure 4A,B). Note that the peak at 48 h was similar in both tendon types, but the levels during treatment differed between them. MMP-13 reacted to the uniaxial treatment similarly (Figure 5A,B). Its highest protein expression was scored at 48 h with a constant increase over time in both tendon tissue types.

### 2.4. Real-Time Tendon Stiffness During Cultivation in the Bioreactor

**Figure 6:**
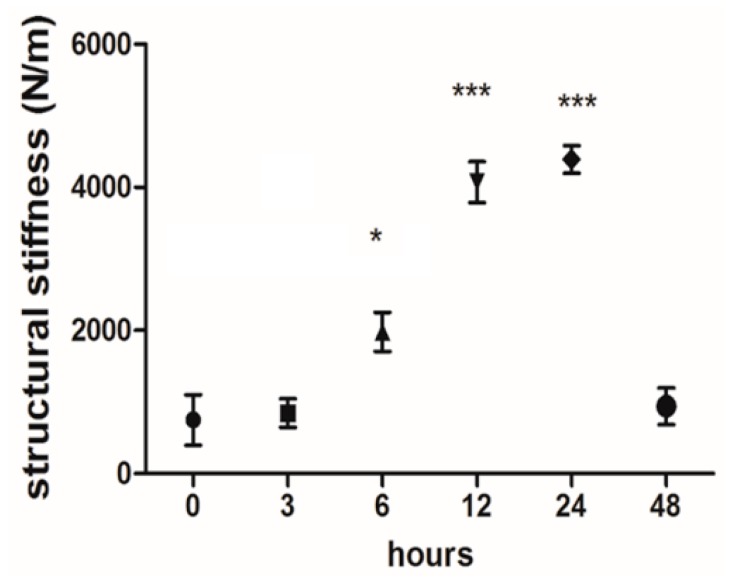
Real-time structural stiffness monitoring during cultivation of the tendon. The average structural stiffness of the samples was plotted during the application of cyclic uniaxial mechanical stimulation. The graph shows the mean stiffness values (forces/elongation) measured at each time interval. The structural reaction force of the tendon tissues after 6 h was significantly higher than at the beginning (1981 ± 272 N/m vs. 755 ± 354 N/m). This trend continues to increase through 12 and 24 h (4.074 ± 285 N/m and 4.391 ± 192 N/m, respectively). The measured force and, therefore, the stiffness, declined in the latter half of the cultivation (941 ± 255 N/m at 48 h). *: *p* ≤ 0.05 and ***: *p* ≤ 0.001.

Our in-house tensile bioreactor system provides real-time information on the reaction forces, which it measures using calibrated load cells. During the experiments, the software visualizes the current forces and displacements and stores the measured data. These real-time data on reaction forces (with respect to the elongations) represent the stiffness of the tissue. Here, this structural stiffness can be obtained by the ratio of the reaction force to the elongation. As shown in Figure 6, the average structural stiffness of the samples was plotted over 0, 3, 6, 24, and 48 h as cyclic uniaxial mechanical stimulation was being applied. The reaction force (and hence the structural stiffness) of the tendon tissues reached a maximum at 24 h and then started to decrease in the latter half of the cultivation period despite the strain remaining constant. This pertains to both types of tissue. 

## 3. Discussion

The basic mechanisms of tendon degeneration and subsequent damage would enable us to treat and even prevent injury to tendons [43].

The gliding part of the tendon is exposed to pressure and shear forces in addition to forces exerted by tension. The formation of fibrocartilaginous tissue in gliding areas counteracts these forces [8]. Ozasa et al. examined physiological changes in the ECM of a gliding area. In their in vitro study, they analyzed the gliding resistance of decellularized tendon scaffolds obtained from flexor digitorum profundus tendons [15,44]. Han et al. demonstrated that the microstructural heterogeneity affected the mechanobiology of native fibrocartilaginous tissue. They demonstrated that the average strain transfer to the proteoglycan-rich area in meniscus was significantly decreased when compared with the fibrous area and conclude that the presence of proteoglycans reduces the mechanical signal that reaches the cells [9]. But most of the available studies, in vitro and in vivo alike, address questions of biomechanics and tissue engineering in traction regions of tendon. In the present study, we analyzed the effect of strain stress on protein expression in cells distributed throughout a heterogeneous tissue containing parallel-running collagen fibers or fibrocartilaginous areas. 

### 3.1. Mechanical Loading of Flexor Tendon

Bioreactors have shown promise as tools for cultivating tenocytes ex vivo. Since tenocytes are mechanosensitive cells, the mechanical loading of tenocytes is critical for their expansion, expression of tenogenic factors, and synthesis of ECM proteins [31,32,45,46]. In in vitro experiments, cells are stretched directly; but at the same time, stretching the tissue seems to require a lower amplitude. Vigorous stretching would overtax the collagen fibers in the ECM and lead to micro-fiber rupture. Tenocytes interact directly with the ECM and collagen fibrils. Repeated, excessive tensing and increased elongation—which seems effective in eliciting the differentiation of mesenchymal stem cells into tenocytes [47,48]—causes increased bowing, kink band growth, and fibril rupture in the ECM [49]. The linear range of tendon in which the collagen fibers react linearly to loading depends on the tendon’s thickness and collagen structure [50]. The linear range offers fairly ideal elastic recovery and can serve as an optimal amplitude for ex vivo analysis of tendons and ligaments. There is some evidence that a strain of up to 4% will cause cross-linkages of collagen fibrils to fail. A low amplitude—less than 1%–2%—on the other hand, is a feature of the toe region, where the forces are absorbed mostly by collagen crimping [51]. As far as other current studies go, the strain we selected, 2.5%, represents a moderate treatment for tenocytes and tendons. We assume that the signs of fatigue found in our model were caused by the high number of cycles combined with the stimulation time. Some studies have suggested that repetitive loading contributes to altering tendon biomechanical properties and to tendon injuries [30,52,53]. Benjamin et al. found that the dynamic modulus was diminished following a long duration of about 1000 cycles at both low and high magnitude loading at 1 Hz [54]. 

Most of the published data are from work done with tenocytes or fibroblasts isolated from ligament or traction tendon; and for the most part, the mechanical test has been uniaxial stretching. The effect of cyclic strain on fibrocartilaginous or gliding tendon has not been studied as much. Additionally, the multiscale progression of tendon damage with respect to the consistency of the fibrocartilage area and the number of cycles has not been fully elucidated. The bioreactor used in the present study allows uniaxial mechanical loadings to be applied both to traction tendon and to the fibrocartilaginous gliding region of tendon. We are able to show that uniaxial force has a different effect on protein expression in cells from different areas of the tendon with heterogeneous structures. Interestingly, uniaxial stretching has a greater impact on GAGs protein expression in the gliding than in the traction regions. The presence of GAG and proteoglycan in the tissue can affect the intensity of the forces which the cells are subjected. This therefore has a profound impact on the calcium response of the cells that are located within this heterogeneous area [9]. Based on a 48-h application, we can confirm that the tissue exhibited a maximum reaction force after 24 h of stimulation. In other words, the tendon is already overtaxed, even by the mechanical loading from the first 24 hours’ stimulation. The histological results of a 24-h stimulation likewise reveal that the tendon cells have reacted to mechanical stimulation and significantly altered their intracellular and extracellular protein expression.

Immunofluorescence staining against Scx showed a continuous increase in Scx protein expression in both traction and gliding areas for up to 24 h. During the prolonged stimulation, up to 48 h, Scx levels significantly dropped to basal levels. This might have been caused by the de-differentiation of tenocytes in the tendon tissue. Other studies have shown that cyclic strain affects the expression of Scx [27,55]. The fact that the translocation of scleraxis into the nuclei is taking place suggests it is active, with a moderate expression of its target genes. Unfortunately, we have little data about Scx translocation into the nuclei. After the first mechanical cycles, we observed an increased quantity of Scx-positive nuclei in both areas that decreased to basal level over the course of day two. It seems that uniaxial mechanical stimulation affects Scx activation in the cells in both traction and gliding areas in a similar way. 

Finding increases in both MMP-1 and MMP-13 in the traction and gliding areas lends support to the other data concerning the overloading model. An increase in structural stiffness was observed during the experiments. One explanation for this strengthening effect may be that collagen fibers have aligned after cyclic loading. The claw-grid structure of collagen leads to an increase in mesh structure density during the tendon elongation. This event may enhance the stiffness of the structure after the first cyclical loading [56]. However, after subsequent loadings, the load-bearing capacity of collagen fibers has been reached, and fiber ruptures can occur. The decrease in tendon structural stiffness we measured on the basis of calibrated load cells in the bioreactor verified these data. This decrease, we may conclude, means that the samples were probably damaged as a result of mechanical stimulation being applied for longer than 24 h and for a high number of cycles. Ros et al. observed microfibril deformation and kinking of collagen fibers after 50 cycles of 2.5% strain, defects that considerably worsened after 300 cycles. In that study, width-spanning discontinuity was monitored at 2.5% and 500 cycles and cyclic strain was applied at 0.5 Hz [57]. In the present study, we observed that uniaxial stretching affects the expression of MMPs in gliding areas more so than it does in traction areas of tendon. This should resonate, because tendon ruptures are frequently located in the gliding part of tendons. Our results show that gliding area and traction area respond differently to fatigue loading. Comparing FLD and patellar tendon in mice, Zuskov et al. were recently able to make the same point [24].

### 3.2. Tissue Cultivation

To maintain the original differentiated cell phenotype in both traction and gliding regions of tendon, we avoided isolating the cells, and we used tissue explants from flexor tendons. Considering that bradytrophic tendon tissue is low-vascularized tissue, it as well as the tenocytes can both adapt to ex vivo conditions for a few hours. Our results clearly showed that the cultivation of 48 h did not have any effect on the cell viability of the tendon tissue. The reason for the high cell viability in the gliding area of the tendon might be the dense cell amount in this area. Connizzo et al., using the in vitro explant tendon culture model, found (upon live/dead imaging) significantly increasing cell death on the third day of cultivation [58]. In our model, we used caspase-3 staining to analyze the onset of cell apoptosis. There were only a few positive cells after 48 h had elapsed. We would therefore assert that tendon explants without the surrounding soft tissue may serve as a suitable ex vivo model for short-duration experiments [59].

### 3.3. Limitations

The bioreactor employed in the present study enabled a thorough assessment of uniaxial stretching of elastic scaffolds seeded with tendon tissue or tenocytes. But there were limitations nevertheless. The present study aimed mainly to compare mature, original cells from the traction and gliding parts of the tendon tissue. Long-interval cultivation of tendon tissue (up to three days) leads to cell death and cell apoptosis. Therefore, the experiment had to be concluded within a short time frame. The large quantity of culture media used in the bioreactor dramatically dilutes the tissue supernatant, so it was not feasible to quantify any released peptides or cytokines in the supernatant.

The mainly used methods for setting data in the present study are based on immunobiological analysis, which are semi-quantitative [60]. To verify the immunohistological data regarding protein expression in tenocytes and ECM, additional molecular biological experiments are needed. The Alcian blue staining used to analyze sGAG has been reported by some studies to yield less reproducible results [61,62]. Nevertheless, in a future study, the sGAG amount in various samples should be confirmed using Safranin-O/Fast Green and verified in tissue extracts using alternative methods. Moreover, in any future experiments, we would suggest distributing the number of amplitudes over a larger time scale to prevent fatigue. 

## 4. Materials and Methods

### 4.1. Tendon Preparation

**Figure 7:**
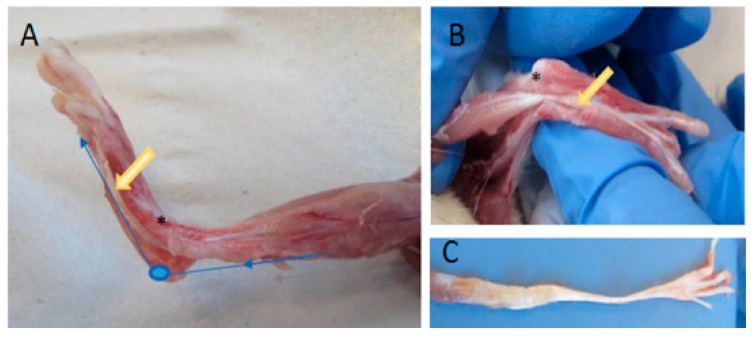
Preparation of a M. flexor digitorum longus tendon. After removing the gastrocnemius muscle, the flexor digitorum longus was exposed (**A**,**B**). The synovium of was cut down and tendon and muscle were mobilized (**B**). For cultivation of the tendon in the bioreactor, the muscle was removed (**C**). The blue line indicates the pathway of the tendon; the yellow arrow, the traction area; an asterisk shows the intrasynovial gliding area.

The tendon tissue was explanted from the flexor digitorum longus muscles (FDL) of 18-week-old Wistar rats (200–250 g) (*n* = 28). Animal procedures were performed according to German legislation for animal protection. The rats’ hind limbs were shaved and disinfected. The skin, fascia, and gastrocnemius muscle were removed (see Figure 7A). After removal of the tendon sheath, the whole FDL structural compound of muscle and tendon was exposed. All FDL tendons used had nearly the same length (3.8 cm) and form. Tendon thickness and width in comparison were modified along the longitudinal axis (see Figure 7C). The muscle tissue was carefully removed and the tendon tissue washed with phosphate-buffered saline (PBS), placed in a 6-cm petri dish and cultivated in 8 mL tenocyte culture media Dulbecco’s modified Eagle’s medium (DMEM) (GIBCO^®^, Thermo Scientific™, Waltham, MA, USA) containing 10% fetal calf serum (FCS), 100 units/mL penicillin and 100 μg/mL streptomycin. Tendon tissues were clamped and incubated in a bioreactor at 37 °C in 95% humidified air and a 5% CO_2_ atmosphere. 

### 4.2. Cultivation of the Tendon Explants in a Bioreactor

**Figure 8:**
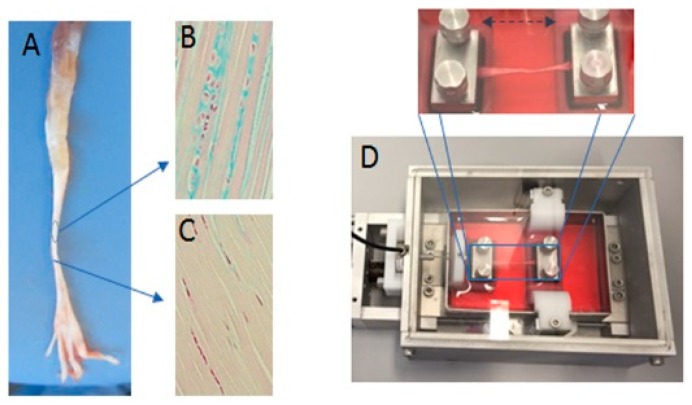
Exemplary images of the tendon and the bioreactor used in the study. (**A**) Tendons of the flexor digitorum longus were extracted from rat legs with two different areas of traction and gliding and stained with an Alcian blue to indicate the varying expression of sulfated glycosaminoglycans (sGAG) in different areas of the tendon (gliding (**B**) and traction (**C**)). (**D**) The image shows the in vitro bioreactor that was used, in which the tendon was fixed between two pads.

The FDL tendon from the right hind limbs was cultivated in a new tissue and cell investigation system (TCIS) bioreactor. To ensure a constant temperature of 37 °C and a constant CO_2_ concentration of about 5%, the bioreactor was placed in an incubator (see Figure 8). For the mechanical stimulation, a periodical elongation of *A* = 0.5 mm (2.5%) with a cycle duration of *T* = 1 s was applied for 3 h. This was followed by a non-stimulation phase for 3 more hours (thus, a 6-h treatment). This pattern was repeated 4 times (for a total number of 43,200 stimulation cycles) within 24 h, or 8 times within 48 h. The reaction force was recorded for the duration of the experiment. The remaining samples served as a control group.

### 4.3. Histological Analysis

After the periodic durational stimulation, the samples were detached, and then underwent a protein histochemical analysis. Tendons were washed with PBS solution and fixed in 4% neutrally buffered formalin solution for at least 18 h at 4 °C. These tendons were divided into three parts: middle, intra- and extra-synovial. The visible fibrocartilaginous area of the intra-synovial part, including gliding tendon and the extra-synovial traction area, were macroscopically different and were highlighted (by embedding) for histological analysis. All sections were placed in paraffin with the same sequence of gliding and traction parts. After dehydration in a graded ethanol series, the samples were oriented during embedding in paraffin. Samples were cut longitudinally at a thickness of 4.5 µm and prepared for histological, immunohistological, or immunofluorescence staining.

### 4.4. Alcian Blue Staining

Alcian blue staining was used to detect sulfated glycosaminoglycans (sGAGs) in fibrocartilage tissues on serial sections. The sections were deparaffinized in 2 × 10 min changes of xylene and rehydrated in a sequential decrease of ethanol (100%, 96%, 80%, and 70% ethanol). The sections were then rinsed in distilled water and incubated in Alcian blue solution for 15 min. After washing under running tap water for 2 min, samples were rinsed in distilled water and then incubated for 4 min in nuclear fast red solution. The samples were washed under running tap water for 1 min and dehydrated using 95% and 100% ethanol and two changes of xylene. Finally, the sections were mounted using Depex (Serva, serving scientists, Heidelberg, Germany). The expression of sGAG was determined by scoring the intensity in at least three different areas per gliding or traction area of a section per tendon by two independent observers who were blinded to the experiment. The mean was then calculated for all values per section. The completely blue-stained ECM has a maximum score of 10 points, whereas sections without positive staining (only bright-pink stained collagen fibers) received a minimum score of 0. 

### 4.5. Immunohistological Staining Analysis 

Routine immunohistochemical procedures were performed on serial sections with antibodies against matrix metalloproteinase MMP-1 and MMP-13. For immunohistochemistry, deparaffinized sections were soaked in 3% hydrogen peroxide (H_2_O_2_) for 15 min at room temperature to inhibit endogenous peroxidase activity. The sections were then washed with Tris-buffered saline (TBS) and blocked with 6% bovine serum albumin (BSA) in distilled water. Afterwards, sections were incubated for 24 h at 4 °C with primary antibodies against MMP-1 (Oncogene, IM35L; 1:500 in Tris-buffered saline) or MMP-13 (Santa Cruz Biotechnology,; sc-412363; 1:150 in Tris-buffered saline). The primary antibody was omitted in the negative control experiments. After being washed for 3 × 5 min in PBS, the sections were incubated with HRP-conjugated secondary antibodies for 30 min (1:400, Agilet DAKO, Santa Clara, CA, USA; E0354 or E0453, respectively) and finally visualized with the AEC-Kit (Invitrogen Corporation, Carlsbad, CA, USA) for 5 min. Afterwards, they were counterstained with hematoxylin. The expression of MMPs was determined via scoring of the intensity and area of the immunohistochemical staining in at least three different areas per gliding or traction area of a section per slids by two independent observers who were blinded to the experiment. We haved used at least four slides for each tendon. Then, a mean was calculated of all values per section. An area with all positively stained cells against MMP-1 or MMP-13 has a maximum score of 10 points, whereas a section without positively stained cells has a minimum score of 0. 

### 4.6. Immunofluoresence Analysis

Routine immunofluorescence procedures were performed on serial sections with antibodies against scleraxis (Scx) and caspase-3. First, the sections were deparaffinized, washed three times with 0.1% Triton X in PBS for 5–10 min, and then three times with Tris-buffer for 5 min. Afterwards, slides were blocked with 1.5% BSA in Tris for 20 min. Immunostaining against Scx (Santa Cruz Biotechnology, Heidelberg, Germany; sc-49352; 1:200 in Tris, 2 h) was used to characterize the differentiated phenotype of the tenocytes. Immunostaining against caspase-3 (oncogene, AM34; 1:500, 1:150 in Tris, 2 h) was used to detect apoptotic cells. Rat placenta was used as a positive control. Alexa Fluor 555 (Life Technologie, A21206) and Alexa Fluor 488 (Life Technologies, A21202; 1:250 in 1.5% BSA in Tris, 2 h) were used subsequently. DAPI staining was used to counterstain the nuclei. Images were made using a Keyence BZ-9000 microscope (Keyence, Osaka, Japan) with a 20× PlanFlour El NA 0.45 Ph1 objective lens and equal exposure times. Fluorescence intensity was investigated by ImageJ software (National Institutes of Health, Bethesda, MD, USA) [63]. We delineated the tendon tissue using the polygon selection tool and measured the area of the selection. Then, the red (for Scx) fluorescence signal was selected with the Color Threshold tool using the “Huang” thresholding method. Afterwards, the mean values of the selected signals were measured as well as the area of the selection. In the end, the background mean was subtracted from the mean values of the red or green fluorescence signal. These resulting values were multiplied with the factor of the area of the fluorescence signal divided by the tendon area. Signals were expressed as relative fluorescence units (RFUs). To analyze the amount of Scx translocated into the nuclei, we counted all of the DAPI positive nuclei, Scx-positive cells, and Scx-overlapped nuclei (violet nuclei). The percentage of Scx-positive nuclei was calculated in all groups by the mean obtained by two independent observers who were blinded to the experiment, counting at least three independent traction or gliding areas per sample section.

### 4.7. On-Time Stiffness

To explore the effect of cyclic loading on whole-tendon stiffness, live measurement of the reaction forces was evaluated. The in-house tensile bioreactor system provides real-time information about the reaction forces, measured using calibrated load cells. Such real-time information about reaction forces represents the stiffness of the tissue. During the experiments, the software visualizes the current forces and displacements and stores the measured data. 

### 4.8. Viability Assay

Cell viability was evaluated by the CellTiter-Blue^®^ Cell Viability Assay (Promega, Madison, WI, USA) for analysis of cell viability of tissues up to two days. We mustered the ability of living cells to convert a redox dye (resazurin) into a fluorescent end product (resorufin) directly after preparation and again after 48 h. The measured fluorescence signal is proportional to the number of viable cells in the tendon. After removal of the tendon sheath, the whole FDL structural was exposed. Both the visible fibrocartilaginous area of the intra-synovial part (including gliding tendon) as well as the extra-synovial area (including traction tendon) were divided into three or four pieces in the equal length (one sample was reserved for positive control). The preparation is borne in PBS, including 100 units/mL penicillin and 100 μg/mL streptomycin. Each sample was placed in one well of a 96 well-plate and incubated with 100 µL culture media until analysis (directly or after 48 h). The positive control was treated with 0.5 mM H_2_O_2_. For the assay, the media supernatant was removed, and the reagent diluted in phenol free medium in a 1:6 ratio. Each 150 μL detection solution was pipetted onto the samples and incubated for 120 min in incubator. From each sample, 70 μL were pipetted to a fresh 96-well plate as a duplicate. Medium diluted reagent without any contact with the tissue cells was used as a blank value. The fluorescence signal was detected at 560 nm excitation and 590 nm emission using a fluorescence microplate reader (Infinite M200, TECAN, Salzburg, Austria).

### 4.9. Statistics

We used six rat tendons per group of each experiment. Each area (gliding or traction) was analyzed in quadruplicate using at least four slides of each tendon. Residuals were analyzed for normal distribution using the Shapiro-Wilk normality test, putting all values in one group. Next, variance homogeneity was tested using the Bartlett test for one-way ANOVA, or the Spearman’s rank correlation test for heteroscedasticity. All data fit these criteria for parametric analysis. Intergroup differences were tested by one-way or two-way ANOVA, followed by Tukey’s post-hoc test. All statistics were performed using GraphPad Prism, version 8.0.0 for Windows, GraphPad Software, San Diego, CA, USA, www.graphpad.com. Data are given as arithmetic means ± SEM. The *p*-values are given as *p* ≤ 0.05, *p* ≤ 0.01, and *p* ≤ 0.001. Asterisks indicate statistical differences in comparison to traction tendon, letters indicate statistical differences in comparison to gliding areas of tendon, and hashtags indicate statistical differences between traction and gliding regions of tendon per time point.

## 5. Conclusions

Understanding the basic mechanisms of chronic tendon degeneration and subsequent injury would enable us to treat and even prevent injury to tendons. In the present study, we have considered the effect of stretching at different cycles and time point in both the intrasynovial gliding part as well as the extrasynovial traction part of the flexor tendon. We used an amplitude just greater that of the toe area to ensure moderate loading of the tendon in the linear phase. 

We observed a heightened intensity of scleraxis up to 24 h of stimulation in both tendon types, whereas MMPs level were increased in both types. In the same time, an increase in structural stiffness, on the basis of calibrated load cells, was observed. During the prolonged stimulation out to 48 h, Scx levels significantly dropped to basal levels and the level of MMPs was increased further, while the decrease in structural stiffness was monitored. This decrease, we may conclude, means that the samples were probably damaged. We assume that the signs of fatigue found in our model were caused by the high number of cycles combined with the stimulation time. The uniaxial loading of tendon affects the tenocytes in traction region and choncrocyte-like cells in the gliding region in a different way. We observed that uniaxial stretching affects the expression of MMPs and sGAG in gliding areas more so than it does in traction areas of the tendon. This should resonate, because tendon ruptures are frequently located in the gliding part of tendons. A combination of mechanical and histological data allows us to improve the conditions for cultivating tendon tissues. To verify the immunohistological data, and to examine and quantify the gene expression or protein level of tenocyte anabolic and catabolic markers, additional molecular biological experiments should be performed.

## Supplementary Materials

Supplementary materials can be found at https://www.mdpi.com/1422-0067/21/8/2925/s1.

## Abbreviations

BAS	Bovine serum albumin
°C	Celsius
CO_2_	Carbon dioxide
COL1	Typ 1 collagen
DMEM	Dulbeccos modified eagles medium
ECM	Extracellular matrix
FCS	Fetal calf serum
h	hours
H_2_O_2_	Hydrogen peroxide
min	Minutes
MMP	Matrix metalloproteinases
PBS	Phosphate buffered saline
SCX	Scleraxis
sGAG	Sulfuted glycosaminoglycans
TBS	Tris-bufferd saline
TIMP	Tissue inhibitors of metalloproteinases
TNMD	Tenomodulin
Tris	Tris (hydroxymethyl) aminomethane
VEGF	Vascular endothelial growth factor
