# Supplementary material for: Impact of Uniaxial Stretching on Both Gliding and Traction Areas of Tendon Explants in a Novel Bioreactor
Source: Int J Mol Sci. 2020 Apr 22;21(8):2925. doi: 10.3390/ijms21082925 (PMC7215532; doi:10.3390/ijms21082925)
Supplement: Supplementary file 1 [file ijms-21-02925-s001.pdf]

Supplementary Materials

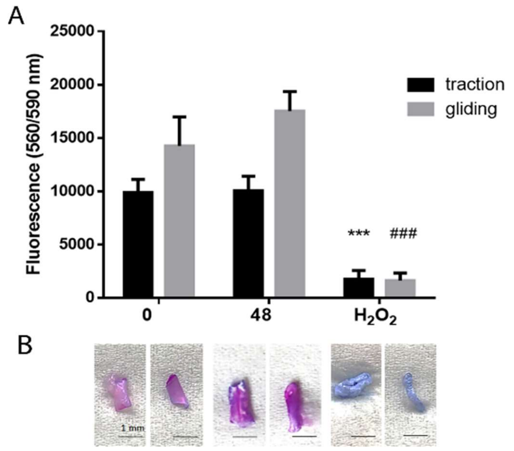

Figure S1. Cell viability of tendon explant during the experiment: To analyze the cell viability of tendon explants after 48 h, we used a Cell-titer-blue® assay, which evaluates the mitochondrial, cytosolic, and microsomal enzyme activity. There was no difference between the cell viability of native tissue directly after preparation and that of the cultivated sample up to 48 h in culture media. Resazurin is dark blue and has little intrinsic fluorescence, which is detected in the positive control, when treated with H_2O_2. During cell activity, Resazurin is reduced to resorufin, which is pink and highly fluorescent. The fluorescence signal was measured in the supernatant (**A**), whereas the tissues demonstrate the absorbed color (**B**).

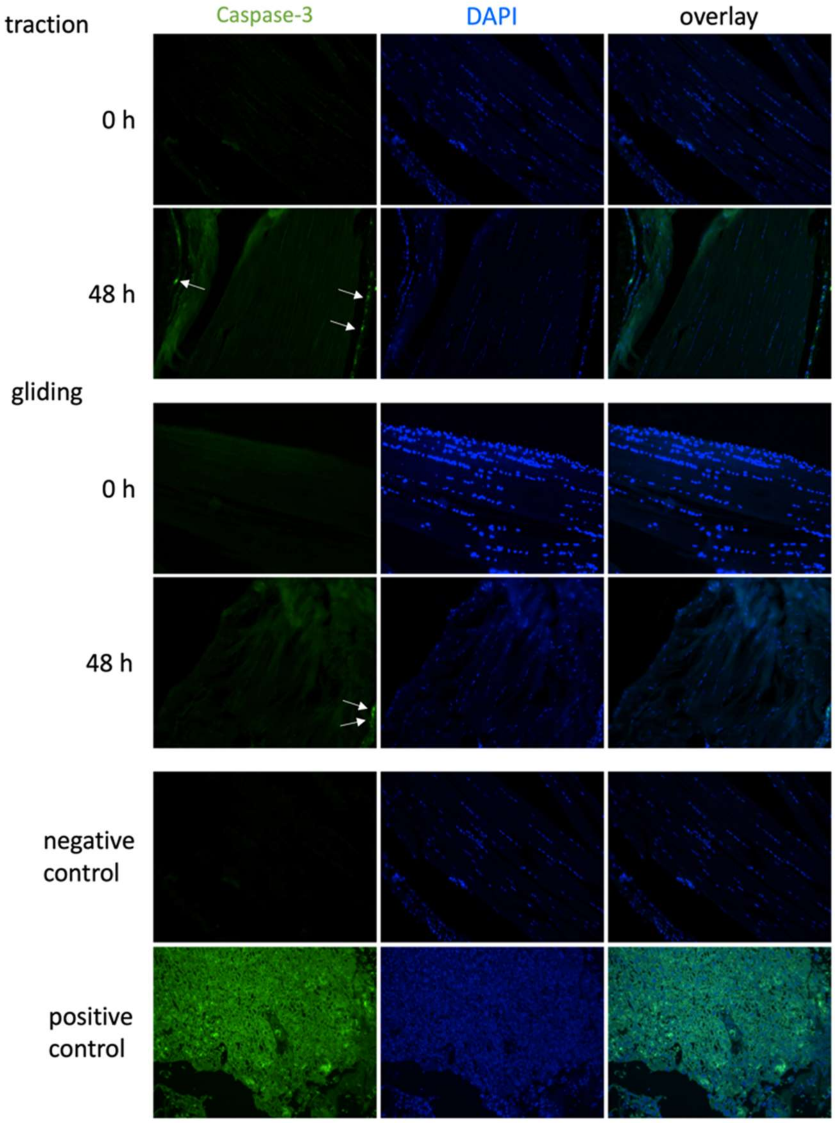

Figure S2. Effect of uniaxial treatment on caspase-3 expression in traction and gliding areas of tendons. The uniaxial treatment leads occasionally to caspase-3 stained cells (indicated by white arrows) in both tendon types after 48 h. In contrast, placental tissue served as a positive control for apoptotic cells, indicated by massive caspase-3 signals. Note that in the negative control, the primary antibody was omitted.
